# The supplementation of a high dose of fish oil during pregnancy and lactation led to an elevation in Mfsd2a expression without any changes in docosahexaenoic acid levels in the retina of healthy 2-month-old mouse offspring

**DOI:** 10.3389/fnut.2023.1330414

**Published:** 2024-01-24

**Authors:** Irena Jovanovic Macura, Ivana Djuricic, Tamara Major, Desanka Milanovic, Sladjana Sobajic, Selma Kanazir, Sanja Ivkovic

**Affiliations:** ^1^Institute for Biological Research “Sinisa Stankovic”, National Institute of Republic of Serbia, University of Belgrade, Belgrade, Serbia; ^2^Faculty of Pharmacy, University of Belgrade, Belgrade, Serbia; ^3^Vinca Institute for Nuclear Sciences, National Institute of Republic of Serbia, University of Belgrade, Belgrade, Serbia

**Keywords:** fish oil, retina, Mfsd2a, RPE, EPA, DHA, pregnancy, lactation

## Abstract

**Introduction:**

During fetal development, the proper development of neural and visual systems relies on the maternal supplementation of omega-3 fatty acids through placental transfer. Pregnant women are strongly advised to augment their diet with additional sources of omega-3, such as fish oil (FO). This supplementation has been linked to a reduced risk of preterm birth, pre-eclampsia, and perinatal depression. Recently, higher doses of omega-3 supplementation have been recommended for pregnant women. Considering that omega-3 fatty acids, particularly docosahexaenoic acid (DHA), play a crucial role in maintaining the delicate homeostasis required for the proper functioning of the retina and photoreceptors the effects of high-dose fish oil (FO) supplementation during pregnancy and lactation on the retina and retinal pigmented epithelium (RPE) in healthy offspring warrant better understanding.

**Methods:**

The fatty acid content and the changes in the expression of the genes regulating cholesterol homeostasis and DHA transport in the retina and RPE were evaluated following the high-dose FO supplementation.

**Results:**

Our study demonstrated that despite the high-dose FO treatment during pregnancy and lactation, the rigorous DHA homeostasis in the retina and RPE of the two-month-old offspring remained balanced. Another significant finding of this study is the increase in the expression levels of major facilitator superfamily domain-containing protein (Mfsd2a), a primary DHA transporter. Mfsd2a also serves as a major regulator of transcytosis during development, and a reduction in Mfsd2a levels poses a major risk for the development of leaky blood vessels.

**Conclusion:**

Impairment of the blood-retinal barrier (BRB) is associated with the development of numerous ocular diseases, and a better understanding of how to manipulate transcytosis in the BRB during development can enhance drug delivery through the BRB or contribute to the repair of central nervous system (CNS) barriers.

## 1 Introduction

The proper development and function of the retina depend on the adequate supply of omega-6 (n-6) and omega-3 (n-3) long-chain polyunsaturated fatty acids (LC-PUFAs) (1, 2). Docosahexaenoic acid (DHA, C22:6n-3), an omega-3 fatty acid, is one of the main retinal structural lipids, comprising up to 50% of the total photoreceptor rod outer segment lipid content (3). The change in the optimal amount of DHA levels can affect the fluidity of the membrane (4) and, consequently, the activity and regeneration of rhodopsin, ultimately affecting phototransduction (2, 4–6). In addition, the proper DHA supply prenatally and early in life is necessary for the proper visual and cognitive functions in the offspring (7–9). Furthermore, the optimal supply of n-3 LC-PUFAs during pregnancy and lactation is associated with a decreased rate of preterm birth (10), reduced risk of pre-eclampsia (11), post-partum depression (12), and the development of allergies (13). In addition, this perinatal and early postnatal period of life is considered particularly susceptible to the effects of environmental factors such as nutrition on beneficial epigenetic changes (14). It was shown that PUFA supplementation can alter epigenetic patterns related to allergic manifestation, and FO consumption was associated with altered histone acetylation in placentas (15). For example, the levels of cord blood T-cell PKCζ could be altered by FO supplementation in an epigenetic manner, affecting the development of allergic inflammation in children (16).

DHA deficiency during pregnancy and lactation affects human retinal development (17, 18), and supplementation with PUFAs throughout pregnancy is recommended as beneficial (19). Mammalian cells lack the ability to synthesize n-6 and n-3 PUFAs *de novo*. Consequently, the fetus relies on maternal transfer through the placenta and subsequent intake through milk and dietary supplements post-birth to accumulate these essential fatty acids. Therefore, it is crucial for the mother to maintain sufficient PUFAs in her diet or through dietary supplements (20). Research has validated a correlation between the concentration of 22:6n-3 in maternal plasma and its placental transfer (21).

Breast milk serves as the main source of PUFAs for newborns, and its PUFA composition is influenced by maternal dietary habits (22). However, several reports have highlighted insufficient n-3 LC-PUFA intake among a significant number of pregnant women in Europe (23–25). Additionally, pregnancy is linked to a notable decline in maternal DHA levels (26–28). Given these current dietary patterns, mothers may struggle to fulfill the heightened fetal DHA requirements. Consequently, prevailing guidelines suggest that pregnant women should aim for a daily intake of 250–500 mg of eicosapentaenoic acid (EPA) and DHA, with at least 200 mg specifically from DHA (29–31). The European Food Safety Authority recommends an extra 100–200 mg of DHA daily (32).

Fish oil (FO) serves as a natural source of n-3 fatty acids (33). Studies have demonstrated the safety and pivotal role of high-dose FO supplementation (up to 5 g/day for 16 weeks) in preventing and managing various diseases in the human population (32). Clinical trials in Denmark, Norway, and Australia involving pregnant women who consumed 2.2–2.7 g of omega-3 fatty acids through fish oil supplements (containing 920 mg, 1,183 mg, and 2.2 g DHA, respectively) revealed positive effects of high-dose FO supplementation (20, 34–36). The extended gestational period observed in the supplemented group was correlated with the DHA concentration ratio in neonatal cord blood. Notably, at 2.5 years of age, children in the fish oil-supplemented group exhibited superior eye and hand coordination scores compared to the control group, which were also in correlation with EPA and DHA in cord blood (20). Similarly, an assessment of the Norwegian infant cohort found that children born to mothers supplemented with FO demonstrated higher mental processing scores at the age of four (37). Additionally, the Australian government has revised its recommendations and, currently, advises pregnant women with low n-3 status to supplement with the augmented dose of n-3 LC-PUFA—specifically, 800 mg DHA and 100 mg EPA per day—primarily to mitigate the risk of preterm birth (38).

However, the impact of high-dose fish oil (FO) supplementation during pregnancy and lactation on the fatty acid composition in the retina and retinal pigmented epithelium (RPE) of offspring in early adulthood remains unclear. Despite the close association between the retinas and RPE (39), they diverge in their requirements for cholesterol and n-3 PUFA homeostasis, potentially responding disparately to FO supplementation during pregnancy and lactation. Moreover, fish oil supplementation has been reported to possess cholesterol-lowering properties in various systems (40–42). Cholesterol is yet another retinal lipid whose changes can affect the activity of rhodopsin, enhancing light activation (43). Given that the retina acquires cholesterol both from blood-borne uptake and local biosynthesis, there is a pressing need to gain a deeper understanding of how dietary treatments impact the transcriptional network of cholesterol-related gene expression.

The retina’s absorption of DHA involves receptors for lipoproteins and adiponectin (1, 44), along with multiple transporters for fatty acids (45). Nevertheless, the primary route for DHA uptake into the retina is likely through the pathway mediated by the major facilitator superfamily domain-containing protein 2a (Mfsd2a) (46–48). Mfsd2a has been identified as a key regulator of blood–organ barrier permeability, encompassing both the blood–brain barrier (BBB) and the blood–retinal barrier (BRB) (49, 50). Mfsd2a-mediated lipid transport is crucial for inhibiting transcytosis (51), and a decrease in Mfsd2a expression is linked to impairments in vascular permeability. However, this reduction in Mfsd2a expression leads to a subsequent increase in vesicle trafficking (enhanced transcytosis) and compromised barriers, all occurring without alterations in endothelial junctions (47, 52). Conversely, studies have demonstrated that heightened Mfsd2a expression can reduce transcytosis (53).

In a previous study, we demonstrated that high-dose fish oil (FO) supplementation during adulthood led to significant alterations in lipid content and the increased expression of genes governing DHA transport (Mfsd2a) and cholesterol homeostasis in the retina and retinal pigmented epithelium (RPE) (54). However, the enduring effects on the retinas and RPE in the offspring resulting from high-dose FO supplementation during pregnancy and lactation remain unknown. To address this, we investigated the phospholipid content in the retinas and RPE, as well as changes in the expression levels of genes involved in DHA transport and cholesterol homeostasis in the retinas and RPE of 2-month-old B6/SJL female mice born to mothers who received high-dose FO supplementation during pregnancy and lactation.

## 2 Materials and methods

### 2.1 Animals

For this study, female B6/SJL mice were utilized. All procedures involving animals adhered to the EU Directive (2010/63/EU) concerning the protection of animals used for experimental and scientific purposes. Approval was obtained from the Ethical Committee for the Use of Laboratory Animals (resolution No. 01–06/13) at the Institute for Biological Research, University of Belgrade. The animal procedures also conformed to the EEC Directive (86/609/EEC) on animal protection, with diligent efforts made to minimize any potential suffering. The mice were housed in standard conditions (23 ± 2°C, relative humidity 60–70%, 12-h light/dark cycle), with regular health check-ups. They had unrestricted access to pelleted commercial rodent chow (see Table 1), available *ad libitum* (AL).

**Table 1:** Pelletized commercial diet content.

Nutrient	% of the total amount
Protein	17.2
Carbohydrate	60.9
Fat	3.7
PUFA/SFA	1.3
n-3/n-6 PUFA	0.05
Fiber	5.6
Ash	7.6

Adequate amount of vitamins and minerals SFA, saturated fatty acids; PUFA, polyunsaturated fatty acids; n-3, omega-3; n-6, omega-6.

### 2.2 Treatment

**Figure 1:**
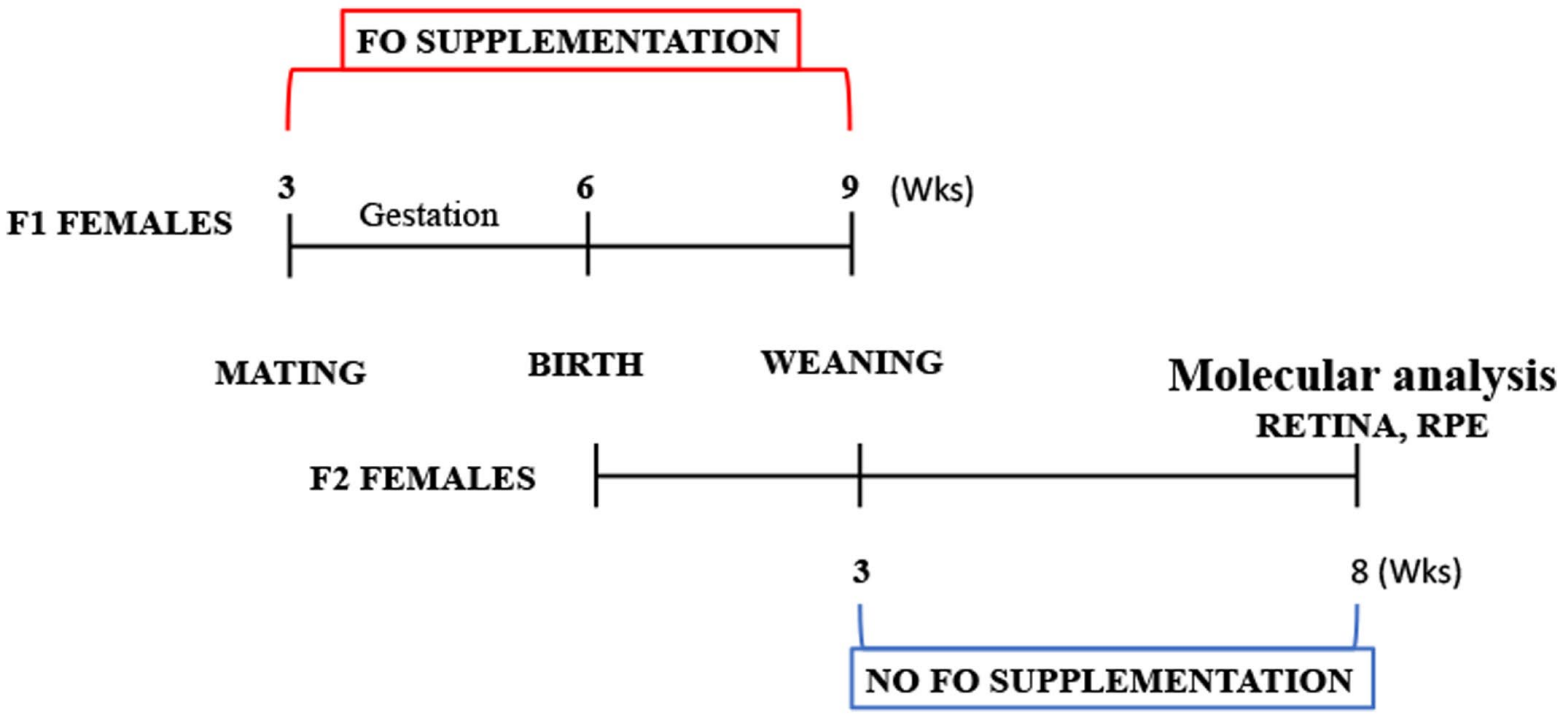
Schematic depiction of the study design.

For the fish oil (FO) treatment, pregnant B6/SJL mice were separated into two groups: the treated group (*n* = 7) received supplementation with commercial fish oil (DietPharm, FidaFarm Croatia), a rich source of omega-3 fatty acids, whereas the control group (*n* = 7) was given the same volume of water as a vehicle. The treated group received 100 μL of fish oil (fatty acid composition in Table 2) daily through oral gavage. The FO treatment spanned 6 weeks, covering both gestation and the lactating period (Figure 1).

**Table 2:** Fatty acid composition of fish oil (% w/w of total fatty acids).

SFA	16:0n	Palmitic	22.90
	18:0n	Stearic	2.23
MUFA	16:1n-7	Palmitoleic	11.90

	18:1n-7	Vaccenic	4.54
n-6	18:2n-6	Linoleic	1.67
	20:3n-6	Dihomo-gama-linolenic	0.29
	20:4n-6	Arachidonic	1.62
	22:4n-6	Adrenic	1.78
n-3	20:5n-3	EPA	25.51
	22:5n-3	DPA	1.82
	22:6n-3	DHA	15.49

SFA, saturated fatty acids; MUFA, mono-unsaturated fatty acids; n-6, omega-6 polyunsaturated fatty acids (PUFA); n-3, omega-3 polyunsaturated fatty acids; EPA, eicosapentaenoic acid; DPA, docosapentaenoic acid; DHA, docosahexaenoic acid.

The chosen dose for this study constituted a high-dose treatment of either DHA or EPA. A daily human dose of 3,000 mg of DHA equates to 50 mg/kg of body weight. The animal equivalent dose (AED) was calculated according to FDA guidelines for species conversion, where AED (mg/kg) = human dose (mg/kg) multiplied by the correction factor for mice (Km). For the human dose of 50 mg/kg, the AED dose amounted to 615 mg/kg. Consequently, with 12 mg DHA and 18 mg EPA per 100 μL, animals were treated with 545.5 mg/kg of DHA and 818.2 mg/kg of EPA daily.

The control group received an equivalent amount of water (100 μL) administered daily through oral gavage during the same period. To maintain experimental purity, we refrained from using other oils as a control due to the potential biological effects of additional components in dietary fish oil, such as omega-3 and omega-6 fatty acids, iodine, furan fatty acids, and antioxidant vitamin E. In order to prevent DHA degradation, one capsule containing 1 mL of FO was used for every 3–4 animals (100 μL per animal) and was administered through oral gavage in under 45 s. Any remaining FO was discarded, and a new capsule was utilized for the subsequent set of animals. After the lactation period, the pups were weaned and provided with regular chow.

Five weeks post-termination of FO supplementation, mice were euthanized at the age of 2 months (Figure 1). Anesthesia was induced (100 mg/kg Ketamidor, Richter Pharma, Wells, Austria, and 16 mg/kg Xulased, Bioveta, a.s. intraperitoneally), followed by perfusion with 50 mL 0.1 M phosphate buffer (PBS) over 30 min before decapitation. Eyes were promptly enucleated, and from the seven eyes, retinas and RPE were isolated and processed for RNA isolation and qPCR. Another set of seven eyes had their retinas and RPE isolated and processed for fatty acid isolation.

### 2.3 Tissue collection

At the time of euthanization, the animals were 2 months old (Figure 1). Mice underwent anesthesia (100 mg/kg Ketamidor, Richter Pharma, Wels, Austria, and 16 mg/kg Xulased, Bioveta, a.s. intraperitoneally). Each animal underwent perfusion with 50 mL 0.1 M phosphate buffer (PBS) for 30 min and was subsequently decapitated. The eyes were enucleated, the optic nerve was severed, and the cornea, lens, and vitreal body were extracted. The retina was carefully peeled off for further analysis. The eyecup, encompassing the RPE, choroid, and sclera (referred to as RPE henceforth), was isolated and utilized for subsequent analysis. All tissue samples were stored at −80°C until RNA and fatty acid isolation. In seven eyes, retinas and RPE were individually isolated and processed for RNA isolation and qPCR. Another set of seven eyes had their retinas and RPE individually isolated and processed for fatty acid isolation. The same animals were used for blood collection to facilitate biochemical analyses.

### 2.4 Retina and RPE fatty acid methyl ester preparation

Retina and RPE total lipids were extracted using chloroform/methanol, following the method outlined by Folch and modified by Kates et al. (55). The conversion of the extracted lipids into fatty acid methyl esters (FAME) was accomplished using 3 M HCl in methanol, as detailed in previous reports (56). The lipids were placed in a glass cuvette, and 1.5 mL of 3 M HCl was added. After mixing, the mixture was heated in a water bath at 85°C for 45 min and then cooled. Hexane (Sigma Aldrich) was introduced for FAME extraction. Following centrifugation for 15 min at 4,000 rpm, the hexane (upper layer) containing the fatty acid methyl esters was transferred into vials using Pasteur pipettes and promptly subjected to analysis.

#### 2.4.1 Gas chromatographic condition

Gas chromatography using Agilent Technologies AGILENT 6890/7890 GC and ChemStation Operation with an FID detector was employed to analyze fatty acid methyl esters (FAMEs). The separation of FAMEs took place on a CP-Sil88 capillary column (a 100-m fused silica capillary column with 0.25 mm internal diameter, coated with 0.2 μm cyano-propyl-polysiloxane as the stationary phase) provided by Supelco (Bellefonte, PA, USA). Chromatographic conditions involved 1 μL injections of the FAME mixture at a split ratio of 20:1. The split inlet conditions included an injector temperature of 250°C, an injector split flow of 20 mL/min, a pressure of 31,623 psi, and a total flow of 24 mL/min. The oven temperature program was initiated at 80°C, increased by 4°C/min up to 220°C (held for 5 min), then increased by 4°C/min up to 240°C, and held at 240°C for 10 min. Helium served as the carrier gas (constant flow of 1.0 mL/min), and nitrogen acted as the makeup gas with a flow of 25 mL/min. The FID detector operated at a temperature of 270°C, and the run time was 55 min. ChemStations was utilized for data collection and analysis, including the identification and quantification of peaks. Chromatographic peak identification was achieved by comparing retention times with an appropriate standard of FAMEs (Supelco FAME Mix, Bellefonte, PA). Quantification relied on the ratio between all peak areas and the corresponding peak, with results expressed as a percentage of individual fatty acids in total fatty acids. The column’s efficiency, expressed as the number of theoretical plates of three standard fatty acids (palmitic, stearic, and oleic), ranged from 362,870 to 510,262. The reproducibility of the response, determined as a percentage of the relative standard deviation (RSD%) for successive measurements of the same reference solution, ranged from 2.3 to 4.6 for the same standard fatty acids.

### 2.5 Real-time time quantitative polymerase chain reaction (qRT-PCR)

#### 2.5.1 RNA isolation and reverse transcription

Total RNA was extracted from the eyes of both control and FO-treated animals (*N* = 5–7 per group) using the TRIzol isolation system, following the manufacturer’s guidelines (Invitrogen Life Technologies, USA). The RNA pellet was dissolved in 20 mL of DEPC water, and the RNA concentration was determined using spectrophotometry. Additionally, RNA integrity was confirmed through 1% agarose gel electrophoresis. Subsequently, 6 mg of total RNA underwent treatment with RNase-free DNase I (Thermo Fisher Scientific, Waltham, MA, USA) and was reverse transcribed in the same tube using a High-Capacity cDNA Archive Kit (Applied Biosystems, USA), following the manufacturer’s protocol. The resulting cDNA was stored at −20°C for future use.

#### 2.5.2 Quantitative real-time RT-PCR (qRT-PCR)

PCR analysis utilized 20 ng of the resultant cDNA in a final volume of 10 μL with RT2SYBR Green qPCR Mastermix (Applied Biosystems). RT-PCR amplifications were conducted in an ABI 7500 thermal cycler (Applied Biosystems) following the default cycling mode (500°C for 30 min, 950°C for 15 min, followed by 40 cycles of 940°C for 60 s, 570°C for 60 s, 720°C for 60 s, and then incubation at 700°C for 10 min). The qRT-PCR results were analyzed using RQ Study add-on software for the 7,000 v 1.1 SDS instrument, with a confidence level of 95% (*p* < 0.05). Quantification was performed using the 2-DDCt method (57), and the change in mRNA levels was expressed relative to the control value. Primer sequences (Vivogen, Serbia) can be found in Table 3.

**Table 3:** Primer sequences for expression studies.

Gene	Orientation	Sequence
*Hmgcr*	F	TTG GTC CTT GTT CAC GCT CAT
	R	TTC GCC AGA CCC AAG GAA AC
*Srebf1*	F	ACG GAG CCA TGG ATT GCA
	R	AAG TCA CTG TCT TGG TTG TTGATGA
*Nr1h2* (*LXRB*)	F	AGC GTC CAT TCA GAG CAA GTG
	R	CAC TCG TGG ACA TCC CAG ATC T
*Abca1*	F	AGG CCG CAC CAT TAT TTT GTC
	R	GGC AAT TCT GTC CCC AAG GAT
*Apoe*	F	GGC CCA GGA GGA GAA TCA ATGA G
	R	CCT GGC TGG ATA TGG ATG TTG
*Mfsd2a*	F R	AGA AGC AGC AAC TGT CCA TTT. CTC GGC CCA CAA AAA GGA TAA T
*Adipor1*	F R	AAG CCA AGT CCC AGG AAC AC CAG TGG GAC CGG TT GC
*Cyp46a1*	F R	TGC AGT ATC TGT CGC AGG TC TAG GTG CTG AAC AGG AGA GG
*Cyp27a1*	F R	CGC TAG TCT CCC TAT GTC ACT ATG C AGC CGA AGG GAA GAG ATG C
*Hprt1*	F	CTC ATG GAC TGA TTA TGG ACA GGA C
	R	GCA GGT CAG CAA AGA ACT TAT AGC C

F, forward primer; R, reverse primer.

### 2.6 Statistical analysis

The Prism program (GraphPad Software) was employed for data analysis. The non-parametric Mann–Whitney *U* test was utilized to compare two experimental groups, given that the data did not adhere to the normal distribution criteria. Statistical significance was established at *p* < 0.05.

## 3 Results

### 3.1 The high-dose FO treatment during pregnancy and lactation altered the fatty acid content in the retinas and RPE of the 2-month-old offspring

**Figure 2:**
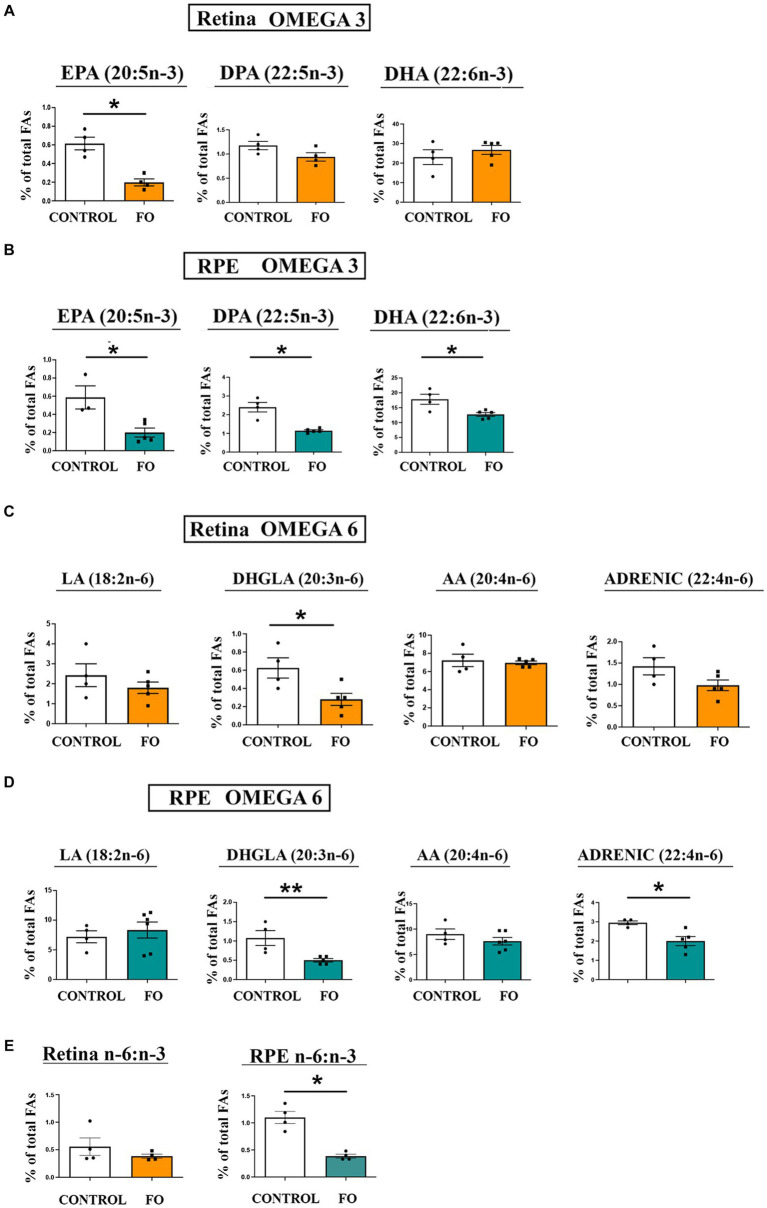
The impact of high-dose fish oil (FO) supplementation on the levels of n-3 and n-6 PUA in the retina and retinal pigmented epithelium (RPE) of both control and FO-supplemented offspring. **(A)** Displays the relative content of n-3 LC-PUFA in the retina, whereas panel **(B)** represents the same in the RPE of 2-month-old control and FO-treated offspring (FO). **(C)** Showcases the relative content of n-6 LC-PUFA in the retina, and **(D)** presents the equivalent in the RPE for both control and FO-treated offspring. The n-6/n-3 LC-PUFA ratio in the retinas and RPE for both groups is depicted in panel **(E)**. The abbreviations denote linoleic acid (LA, 18:2n-6), dihomo-gamma-linoleic acid (DHGLA, 20:3n-6), arachidonic acid (AA, 20:4n-6), adrenic acid (22:4n-6), eicosapentanoic acid (EPA, 20:5n-3), docosapentaenoic acid (DPA, 22:5n-3), and docosahexaenoic acid (DHA, 22:6n-3). The data are presented as mean ± SEM. *p < 0.05, **p < 0.01.

We evaluated the alterations in the lipid composition, specifically n-6 and n-3 fatty acids, resulting from high-dose fish oil (FO) supplementation in the retinas and retinal pigmented epithelia (RPE) of the 2-month-old offspring (Figures 2A–E). In the offspring’s retinas, there was a significant reduction in EPA levels (3.1-fold decrease, Figure 2A). Conversely, the levels of DHA and DPA remained unchanged (Figure 1A). However, in the RPE, all analyzed n-3 fatty acids, including EPA (2.95-fold decrease), DPA (2.18-fold decrease), and DHA (1.38-fold decrease), exhibited a reduction (Figure 2B). Regarding n-6 fatty acids, there was a decrease in dihomo-gamma-linoleic acid (DHGLA) levels (55%) in the FO-supplemented retinas (Figure 2C). In the RPE, along with a decrease in DHGLA levels (53%), there was a significant reduction in adrenic acid levels (32% decrease) (Figure 2D). While the n-6/n-3 ratio in the retina remained unchanged in FO-treated and control offspring, a significant decrease was observed in the RPE (65% decrease, Figure 2E).

### 3.2 The impact of fish oil treatment on the overall polyunsaturated fatty acids, mono-unsaturated fatty acids, and saturated fatty acids in both the retina and retinal pigmented epithelium

Given that the analysis of fish oil (FO) content revealed the presence of fatty acids other than essential PUFAs, particularly palmitic, palmitoleic, and oleic (Table 2), we investigated whether FO intake alters the levels of saturated fatty acids (SFAs) and mono-unsaturated fatty acids (MUFAs) in both the retinas and RPE. In the retinas of the FO-supplemented offspring, the sole observed change was a decrease in oleic acid levels (34%) (Table 4). However, in the FO-supplemented RPE, there was an increase in the levels of palmitic (12%), palmitoleic (91%), and oleic acid (41.5%) (Table 4). Despite this, total SFA and PUFA remained unchanged after FO treatment in both the retinas and RPE. Notably, n-3 levels were significantly reduced in the RPE (28%), whereas total MUFA showed an increase in the RPE exclusively (42%).

**Table 4:** SFA, MUFA, and PUFA in the retinas and RPE of control and FO-supplemented mice.

Fatty acid %	Retina	Retina FO	RPE	RPE FO
Palmitic acid (16:0)	19.93 ± 0.22	20.9 ± 0.28	18.58 ± 0.29	20.95 ± 0.724^b^
Stearic acid (18:0)	26.13 ± 1.84	25.74 ± 1.52	22.65 ± 0.56	19.57 ± 0.89
SFA	46.5 ± 1.97	47 ± 1.61	41.7 ± 0.71	41.2 ± 0.75
Palmitoleic acid (16:1n-7)	0.45 ± 0.06	0.34 ± 0.05	1.17 ± 0.26	2.25 ± 0.25^b^
Oleic acid (18:1n-9)	9.87 ± 0.54	6.48 ± 0.37^a^	10 ± 0.72	14.15 ± 0.65^c^
Vaccenic acid (18:1n-7)	1.53 ± 0.21	1.58 ± 0.18	2.03 ± 0.18	2.45 ± 0.07
MUFA	10.7 ± 1.23	8.46 ± 0.56	13.2 ± 0.81	18.95 ± 0.90^c^
n-6	12.2 ± 1.12	10 ± 0.02	22.5 ± 1.83	18.9 ± 0.08
n-3	24.8 ± 3.76	27.1 ± 2.79	20.2 ± 1.66	14.6 ± 0.59^c^
PUFA	32.7 ± 3.29	35.5 ± 2.39	34 ± 2.19	29.6 ± 0.935

SFA, saturated fatty acids; MUFA, mono-unsaturated fatty acids; n-6, omega-6 polyunsaturated fatty acids (PUFA); n-3, omega-3 PUFA. Values are presented as mean ± SEM. Significantly different from the control retina.

^a^*p* < 0.05. Significantly different from the control RPE: ^b^*p* < 0.05, ^c^*p* < 0.001.

### 3.3 The high-dose FO supplementation during pregnancy and lactation resulted in an elevation of Mfsd2a expression in both the retinas and RPE of the 2-month-old offspring

**Figure 3:**
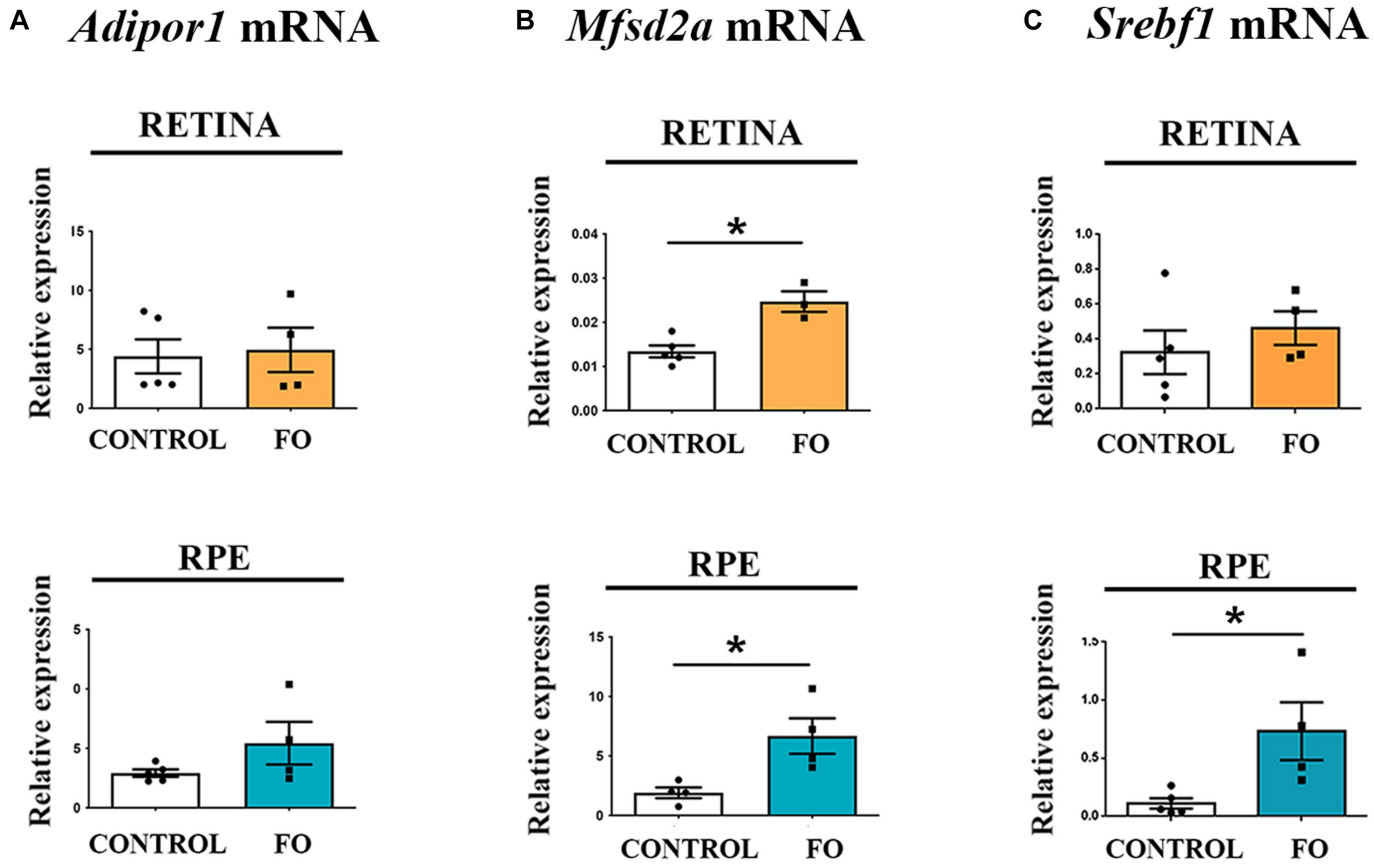
The expression levels of *Mfsd2a* were influenced by high-dose fish oil (FO) supplementation, whereas *Adipor1* levels remained unaffected in the retina and retinal pigmented epithelium (RPE) of 2-month-old offspring. **(A)** Real-time polymerase chain reaction (RT-PCR) was utilized to analyze the alterations in *Adipor1* expression in the retina (yellow) and RPE (blue). **(B)** RT-PCR was employed to assess changes in *Mfsd2a* expression in the retina (yellow) and RPE (blue). **(C)** RT-PCR was used to examine changes in *Srebf1* expression in the retina (yellow) and RPE (blue). The data are presented as mean ± SEM. *p < 0.05.

Two specific proteins, Mfsd2a (48) and the adiponectin receptor 1 (Adipor1) (58), have been proposed as facilitators in the transport of DHA. Mfsd2a is a transporter highly specific for DHA, with lower specificity for palmitic and oleic acids. Adipor1 has recently been identified as a novel DHA transporter crucial for the proper functioning and maintenance of photoreceptors. The expression levels of *Adipor1* were unaffected by the FO treatment in both the retinas and RPE (Figure 3A). In contrast, the expression levels of *Mfsd2a* significantly increased in both the retina (Figure 2B, 84% increase) and the RPE (Figure 3B 3.5-fold increase) following FO supplementation.

Sterol regulatory element-binding protein 1-c (Srebp1-c) is among the suggested targets of Mfsd2a expression (59). Another function of Srebp1-c is to enhance the transcription of genes regulating fatty acid synthesis (60). However, contrary to the reported decrease in *Srebf1* in adult animals supplemented with FO associated with the increase in *Mfsd2a* (54), the expression levels of *Srebf1* remained unchanged in the retinas (Figure 3C) and increased in the RPE of FO-supplemented offspring (6.5-fold increase, Figure 3C).

### 3.4 The high-dose FO supplementation during pregnancy and lactation had opposite effects on the expression levels of *Hmgcr* in the retinas and RPE

**Figure 4:**
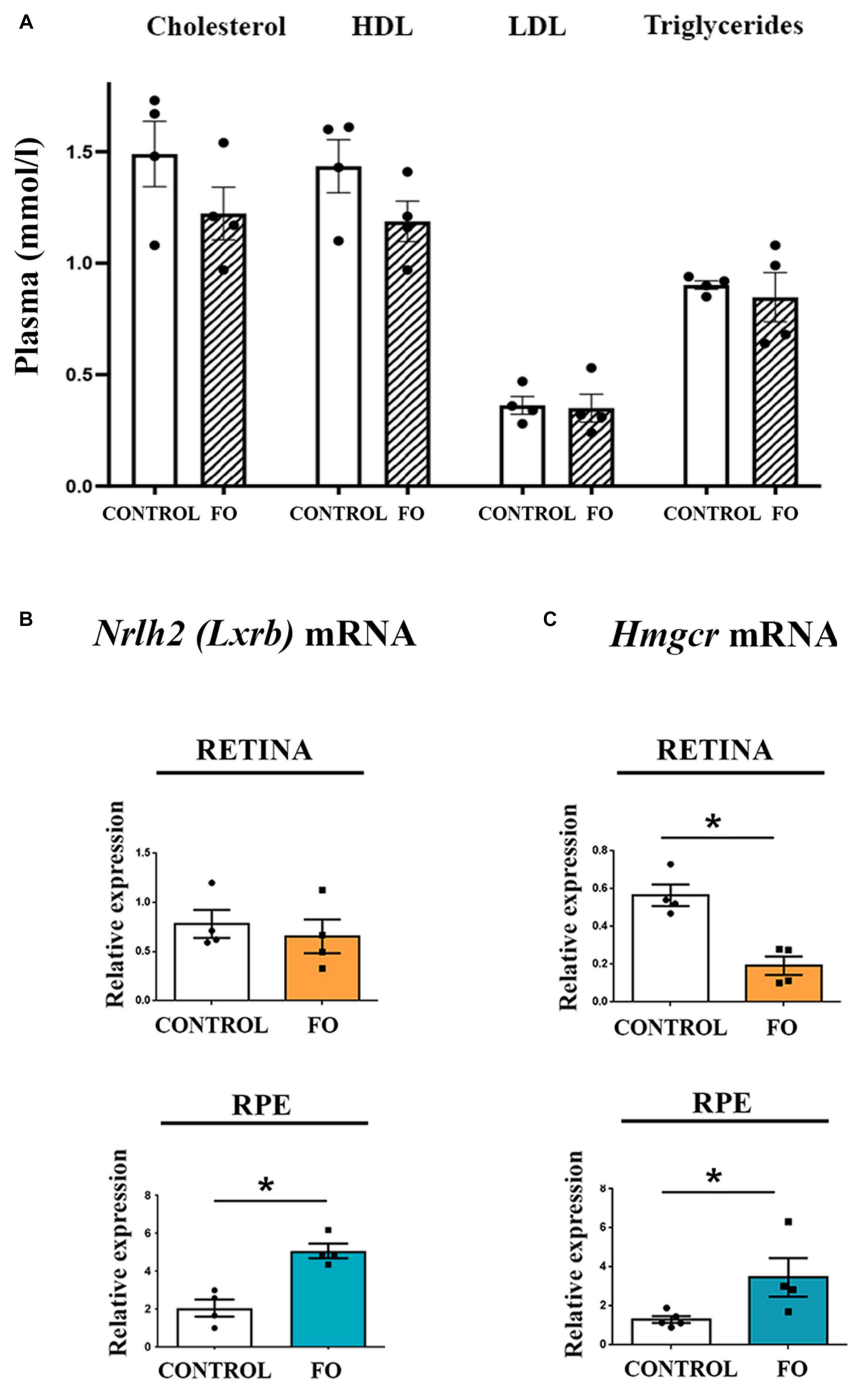
Examination of cholesterol levels in serum and the expression of genes regulating cholesterol synthesis in the retina and retinal pigmented epithelium (RPE) in control and fish oil (FO) supplemented mice. **(A)** Biochemical analyses of cholesterol, HDL, LDL, and triglyceride levels (mmol/l) in the plasma of control and FO-treated mice. Expression levels of *Nr1h2* (*LXRB*) **(B)** and *Hmgcr* **(C)** were assessed in the retina (yellow) and RPE (blue) of control and FO-supplemented offspring using real-time polymerase chain reaction (RT-PCR). The data are presented as mean ± SEM. *p < 0.05.

Fish oil (FO) supplementation is known for its ability to lower cholesterol, yet the biochemical analyses of serum from 2-month-old FO-supplemented offspring revealed no differences in the levels of cholesterol, HDL, LDL, and triglyceride (Figure 4A). Considering that analyses of retinas from Mfsd2a^−/−^ animals showed altered expression of genes regulating cholesterol and fatty acid synthesis (59, 61), we investigated whether FO supplementation affected the expression levels of genes regulating cholesterol synthesis. Specifically, we examined liver X receptor beta *Nr1h2* (*LXRB*) (62) and endoplasmic reticulum-bound 3-hydroxy-3-methylglutaryl-coenzyme-A reductase (*Hmgcr*) expression levels (63). The qRT-PCR analysis revealed that FO supplementation had no effect on the expression levels of *Nr1h2* (*LXRB*) in the retinas but induced a 2.48-fold increase in its expression in the RPE (Figure 4B). Simultaneously, the expression levels of *Hmgcr* decreased in the retinas (65% decrease) but increased in the RPE (2.65-fold increase) following FO treatment (Figure 4C).

### 3.5 The high-dose FO supplementation during pregnancy and lactation modified the expression levels of genes involved in regulating cholesterol transport

**Figure 5:**
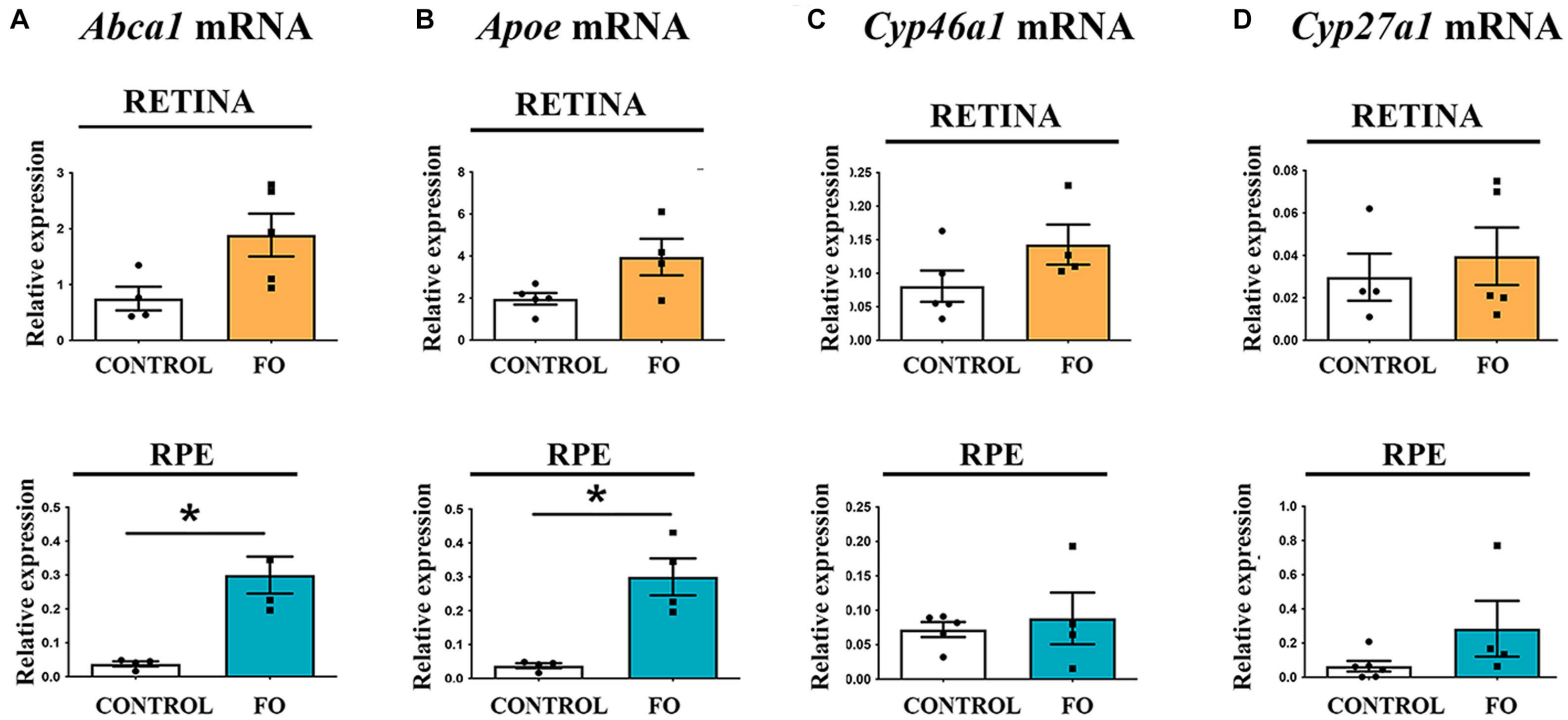
Gene expression profiles associated with cholesterol transport and elimination in the retinas and RPE of both control and FO-supplemented mice. The analysis focused on the expression levels of *Apoe* **(A)**, *Abca1* **(B)**, *Cyp46a1* **(C)**, and *Cyp27a1* **(D)** in the retina (highlighted in yellow) and RPE (highlighted in blue). Real-time polymerase chain reaction (RT-PCR) was employed for this assessment. The data are presented as mean ± SEM, and statistical significance is denoted by * when *p* < 0.05.

We investigated the impact of FO supplementation on cholesterol turnover. Cells efficiently recycle cholesterol through an apolipoprotein-dependent cascade, with apolipoprotein E (ApoE) being the most abundant member (64). The lipidation process is facilitated by the ATP-binding cassette transporter A1 (ABCA1), located in plasma membranes, which expels cholesterol and phospholipids from the cells (64). qRT-PCR analyses indicated that FO supplementation had no effect on *Apoe* and *Abca1* expression levels in the retinas (Figures 5A,B). However, in the RPE of FO-supplemented offspring, the expression levels of *Abca1* and *Apoe* transporters were significantly higher compared to the controls (8-fold and 2.85-fold, respectively) (Figures 5A,B).

Excess cholesterol is primarily eliminated by converting it into 24(S)-hydroxycholesterol (24S-OHC) through the enzyme cholesterol 24-hydroxylase (CYP46A1), predominantly found in the brain (65), and into 27-hydroxycholesterol through the enzyme cholesterol 27-hydroxylase (CYP27A1), a ubiquitous cholesterol 27-hydroxylase (66). Additionally, cytochrome (CYP27A1 and CYP46A1) levels regulate LXR. The qRT-PCR analysis revealed that FO supplementation during pregnancy and lactation had no impact on the expression levels of cholesterol degradation genes *Cyp27a1* and *Cyp46a1* in both retinas or RPE in the offspring (Figures 5C,D).

## 4 Discussion

Scientists and clinicians recommend supplementing with FO, rich in DHA and EPA, during pregnancy and lactation to support the proper development of neural and visual systems. High-dose omega-3 supplementation for pregnant women is strongly advocated (20, 34–36, 39). In this study, we examined the long-term effects of high-dose FO supplementation in healthy pregnant and lactating WT mice on the retinas and RPE of 2-month-old offspring (early adulthood). The key findings include a significant increase in Mfsd2a expression, a primary DHA transporter, in both the retinas and RPE, as well as notable changes in lipid profiles and expression levels of cholesterol metabolism-related genes in both tissues.

FO supplementation during pregnancy and lactation led to persistent changes in fatty acid content, even 5 weeks after supplementation cessation. In the retinas, there was a significant decrease in EPA levels (3.1-fold decrease) among n-3 LC-PUFAs, whereas DHA levels remained unchanged, ensuring proper photoreceptor function. On the other hand, in the RPE, all n-3 LC-PUFAs—EPA, DPA, and DHA—experienced significant decreases (2.95-fold, 2.18-fold, and 1.38-fold, respectively). These results can be explained by the significant lag between the end of the supplementation and the time of analysis (5 weeks). The retinas and RPE of the offspring were exposed to high levels of PUFA supplementation throughout the development, followed by the abrupt termination of the supplementation 3 weeks after birth, coinciding with the termination of lactation and subsequent weaning. The offspring were then switched to commercial chow with a substantially lower content of n-3 PUFAs. It is possible that the offspring that were exposed to the FO supplementation developed a different baseline for the n-3PUFA content and that the period between the end of the FO supplementation and analysis can be perceived as n-3 deprivation. The results suggest a potential period of n-3 deprivation in the offspring. Similarly, in the brains of the offspring that were FO-supplemented pre- and post-natally (67), a continual gradual decline in the levels of DHA was observed over the 8 weeks after the termination of the FO supplementation. The decline in the levels of DHA was similar to our findings in the RPE, while the DHA homeostasis in the retina was not disrupted. At the same time, the increased levels of DPA in the brain were not in correlation with the observed decrease in the levels of DPA in the RPE. These findings suggest a more plastic nature of the RPE considering its physiological role as a “gate-keeper” (68).

Recent studies suggest that EPA could serve as a readily available precursor for DHA synthesis in neuronal tissues, especially in diets enriched with ALA but deficient in DHA (69). The protective benefits of EPA supplementation, however, depend on its conversion to DHA through elongation and desaturation processes in neuronal cultures (70). Typically, elongase and desaturase activities are lower in the brain compared to the liver, where the primary source of brain and retinal DHA is synthesized from circulating ALA (71–73). While it is improbable for neuronal enzymatic activities to be solely responsible for high DHA levels in the retina, they might play a role in maintaining DHA levels in photoreceptors during transient decreases in plasma DHA. These enzymes could also be involved in responding to minor local changes in DHA levels in photoreceptor phospholipids. As DHA is released from these lipids, it activates the retinoid X receptor (RXR) and the ERK/MAPK pathway for photoreceptor protection (74, 75). Neuronal fatty acid elongase and desaturase enzymes may be activated to synthesize and replenish DHA in phospholipids, possibly from EPA, accounting for its decrease.

The impact of FO treatment on n-3 PUFAs in the RPE was more pronounced than in the retinas, leading to a significant decrease in all analyzed n-3 fatty acids—EPA, DPA, and DHA (2.95, 2.18, and 1.38-fold decrease, respectively). The protective role of RPE in maintaining retinal homeostasis could be one of the reasons for these changes, although other mechanisms cannot be ruled out. In a previous study, we demonstrated that high-dose fish oil (FO) supplementation maintained for 3 weeks in 3-month-old mice (adulthood) significantly altered n-3 PUFAs in the retinas and RPE but that the levels of DHA were also unaltered (54). These results confirm that the rigorous regulation of DHA homeostasis in the retina is necessary for the proper functioning of photoreceptors, regardless of the age of the supplementation.

FO supplementation also influenced n-6 PUFA levels, resulting in decreased DHGLA in the retinas and DHGLA and adrenic acid in the RPE. A similar decrease in DHGLA in the RPE was observed in adult animals that were supplemented with FO for a short period of time (3 weeks) (54). Although the n-6 PUFA changes were relatively small, they contributed to an increased n-6/n-3 ratio in the RPE of 2-month-old offspring, while the n-6/n-3 ratio in the retinas remained unaltered. These results strongly suggest that it would be beneficial to maintain the levels of FO supplementation in the late postnatal stages and in adolescence.

Notably, high-dose FO supplementation during pregnancy and lactation elevated *Mfsd2a* expression 5 weeks post-treatment (Figure 2). Increased *Mfsd2a* levels in the retina may contribute to DHA homeostasis, maintaining lipid saturation and cell membrane fluidity (48). In addition, the increase in the expression of the DHA transporter, *Mfsd2a*, was observed both in the adult supplementation and in the offspring that was supplemented during development (54) suggesting that FO supplementation is responsible for this increase, although it is possible that the mechanisms involved in the upregulation of *Mfsd2a* differ depending on the time of the supplementation.

Mfsd2a is specifically expressed in microvessels of the CNS that constitute the blood–brain barrier (BBB), playing crucial roles in both the formation and functioning of the BBB (43, 44). Studies in mice have shown that Mfsd2a expression begins at embryonic day 13.5 (E 13.5) in the BBB, which becomes functional by E15.5 (47). In Mfsd2a knockout (KO) animals, the absence of Mfsd2a resulted in a leaky BBB during embryogenesis, neonatal development, and adulthood without structural vascular abnormalities. Similarly, recent findings highlighted that during the formation of the blood–retinal barrier (BRB), functional tight junctions are present early in vessel ingression, but transcytosis is not yet suppressed (50). This contributes to the leakiness of the retinal vasculature during development, and the gradual suppression of transcytosis in BRB endothelial cells is required for the functional BRB to be established (50). Notably, Mfsd2a expression levels correlate with the functional formation of the BRB by regulating transcytosis. In addition, Mfsd2a deficiency leads to increased transcytosis and incomplete formation of a functional BRB, while premature suppression of transcytosis accelerates BRB development, indicating a time shift in impermeability during development (50).

Regulation of Mfsd2a expression may involve the Srebp pathway, a signaling pathway highly increased in the eyes of Mfsd2a knockout mice (76). Previous research has shown that the expression of *Srebf1* is upregulated concurrently with the decrease in *Mfsd2a* expression in 4-month-old 5xFAD retinas (77), and similarly, it is downregulated in parallel with the increase in *Mfsd2a* expression (54). However, in 2-month-old FO-supplemented offspring, where *Mfsd2a* expression levels were increased, Srebp-1-c levels were also elevated (Figure 2). Srebf1 expression is regulated by LXR, and Srebf1 promoter contains two LXR-responsive elements (LXREs). Interestingly, EPA and DHA have been shown to inhibit the LXR/RXR heterodimer binding to the LXREs in the Srebp-1-c promoter, decreasing Srebp-1-c mRNA levels (78). Therefore, the simultaneous upregulation of *Nr1h2* (*LXRB*) and the downregulation of n-3 PUFAs may contribute to the increased expression levels of *Srebf1* in the RPE.

There have been reports of a close interaction between proteins regulating cholesterol homeostasis and Mfsd2a. Our findings demonstrated increased Lxrβ expression in the RPE of 2-month-old offspring, suggesting an additional mechanism for regulating *Mfsd2a* expression. For example, treatment with the Lxr agonist T0901317 was shown to increase Mfsd2a expression in mice (76), and chromatin immunoprecipitation sequencing (ChIPseq) and gene array studies revealed Lxrβ binding sites in the mouse Mfsd2a intron (79). The upregulation of *Nr1h2* (*LXRB*) could, in turn, enhance the expression of *Hmgcr*, a key factor in cholesterol synthesis, as was observed in the RPE of 2-month-old FO-supplemented offspring (Figure 3).

As the lipid composition of CNS endothelial cells, particularly cholesterol content, plays a crucial role in regulating transcytosis and barrier permeability (51) the role of Mfsd2a as a lipid transporter delivering docosahexaenoic acid (DHA) into the brain (46) is highlighted. The proposed mechanism suggests that the inhibition of caveolae formation and subsequent suppression of transcytosis is maintained through the displacement of cholesterol with DHA and Cav-1 in the membrane (80). Consequently, the upregulation of genes involved in cholesterol synthesis in the RPE may act as a compensatory mechanism to maintain cholesterol homeostasis in the retina. However, the analysis of cholesterol content in the retinal endothelial cells of FO-supplemented offspring is currently lacking. Additionally, unraveling the exact role of proteins responsible for cholesterol synthesis in the regulation of Mfsd2a expression and transcytosis requires further studies.

There is a possibility that omega-3 regulates the expression of *Mfsd2a* through the activation of the Wnt signaling pathway. Disrupted Wnt signaling, as observed in mice lacking LRP5 or Norrin, led to an increase in retinal vascular leakage and, notably, demonstrated heightened transcytosis (81). The Wnt signaling pathway was shown to directly govern the transcription of Mfsd2a in a β-catenin-dependent manner (81), and recent research revealed that DHA supplementation is able to enhance Wnt signaling in a Wnt3a-dependent manner in human-induced pluripotent stem cell-derived neural progenitor cells (NPCs) (82). However, a more in-depth understanding of the interplay between Wnt signaling and omega-3 fatty acids is crucial for comprehending the mechanisms through which FO supplementation can regulate Mfsd2a expression during development.

## 5 Conclusion

The understanding of numerous diseases has been significantly advanced through the analysis of gene expression. While per-gene protein-to-mRNA ratios provide rough estimates of absolute protein abundances across genes, their ability to gauge changes in protein abundance for the same gene across samples is limited and relies on the extent of post-transcriptional, translational, and post-translational regulatory events during tissue development and homeostasis (83). Importantly, the integration of transcriptomic and proteomic data offers additional insights into the principles of gene expression control that cannot be gleaned from either type of data alone. Confirming the importance of transcriptomic analysis, nine databases have been identified that offer ocular transcriptome data from various developmental stages and diverse healthy and diseased ocular tissues. As a result, these databases contribute to the deepening of our knowledge about the molecular mediators involved, facilitate the formation of hypotheses, and assist in identifying novel diagnostic and therapeutic targets for a range of ocular diseases (84).

BRB dysfunction is a pathological characteristic observed in various ocular diseases (85–89). Thus, enhancing our understanding of ways to modulate transcytosis in BRB/BBB during development has the potential to enhance central nervous system (CNS) and retinal drug delivery by facilitating the transport of cargos of different sizes, ranging from compounds less than 1 kDa to large macromolecules. Moreover, targeting genes that regulate transcytosis could be a strategy for repairing CNS barriers in neurodegenerative diseases.

In conclusion, in the case of normal development, the recommended high-dose omega-3 (FO) supplementation may have enduring effects on lipid content and gene expression in the retina and retinal pigment epithelium (RPE) of the offspring. The findings from this study indicate that FO supplementation should be continued or gradually tapered after the lactation period.

## Data availability statement

The original contributions presented in the study are included in the article/Supplementary material, further inquiries can be directed to the corresponding author.

## Ethics statement

The animal study was approved by Ethical Committee for the Use of Laboratory Animals (resolution No. 01-06/13) of the Institute for Biological Research, University of Belgrade. The study was conducted in accordance with local legislation and institutional requirements.

## Author contributions

IM: Visualization, Writing – original draft, Writing – review & editing, Data curation, Formal analysis, Methodology. ID: Formal analysis, Methodology, Visualization, Writing – review & editing. TM: Methodology, Writing – original draft, Writing – review & editing. DM: Formal analysis, Methodology, Visualization, Writing – original draft. SS: Funding acquisition, Writing – review & editing. SK: Funding acquisition, Writing – review & editing. SI: Conceptualization, Supervision, Visualization, Writing – original draft, Writing – review & editing.

## References

[1] SanGiovanni JP Chew EY. The role of omega-3 long-chain polyunsaturated fatty acids in health and disease of the retina. Prog Ret Eye Res. (2005) 2005:87–138. doi: 10.1016/j.preteyeres.2004.06.002 15555528

[2] Sinclair AJ. Docosahexaenoic acid and the brain- what is its role? Asia Pac J Clin Nutr. (2019) 28:675–88. doi: 10.6133/apjcn.201912_28(4).0002, PMID: 31826363

[3] Rotstein NP Politi LE German OL Girotti R. Protective effect of docosahexaenoic acid on oxidative stress-induced apoptosis of retina photoreceptors. Invest Ophthalmol Vis Sci. (2003) 44:2252–9. doi: 10.1167/iovs.02-0901, PMID: 12714668

[4] Querques G Forte R Souied EH. Retina and Omega-3. J Nutr Metab. (2011) 2011:1–12. doi: 10.1155/2011/748361, PMID: 22175009 PMC3206354

[5] Litman BJ Niu SL Polozova A Mitchell DC. The role of docosahexaenoic acid containing phospholipids in modulating G protein-coupled signaling pathways: visual transduction. J Mol Neurosci. (2001) 16:237–42. doi: 10.1385/JMN:16:2-3:237, PMID: 11478379

[6] Bennett MP Mitchell DC. Regulation of membrane proteins by dietary lipids: effects of cholesterol and docosahexaenoic acid acyl chain-containing phospholipids on rhodopsin stability and function. Biophys J. (2008) 95:1206–16. doi: 10.1529/biophysj.107.122788, PMID: 18424497 PMC2479576

[7] Innis SM. Dietary (n-3) fatty acids and brain development. J Nutr. (2007) 137:855–9. doi: 10.1093/jn/137.4.855, PMID: 17374644

[8] Shulkin M Pimpin L Bellinger D Kranz S Fawzi W Duggan C. N-3 fatty acid supplementation in mothers, preterm infants, and term infants and childhood psychomotor and visual development: a systematic review and Meta-analysis. J Nutr. (2018) 148:409–18. doi: 10.1093/jn/nxx031, PMID: 29546296 PMC6251555

[9] Lauritzen L Carlson SE. Maternal fatty acid status during pregnancy and lactation and relation to newborn and infant status. Matern Child Nutr. (2011) 2:41–58. doi: 10.1111/j.1740-8709.2011.00303.x PMC6860497 21366866

[10] Salvig JD Lamont RF. Evidence regarding an effect of marine n-3 fatty acids on preterm birth: a systematic review and meta-analysis. Acta Obstet Gynecol Scand. (2011) 90:825–38. doi: 10.1111/j.1600-0412.2011.01171.x, PMID: 21535434

[11] Burchakov DI Kuznetsova IV Uspenskaya YB. Omega-3 long-chain polyunsaturated fatty acids and preeclampsia: trials say "no," but is it the final word? Nutrients. (2017) 9:1364. doi: 10.3390/nu9121364, PMID: 29244779 PMC5748814

[12] Lin PY Chang CH Chong MF Chen H Su KP. Polyunsaturated fatty acids in perinatal depression: a systematic review and Meta-analysis. Biol Psych. (2017) 82:560–9. doi: 10.1016/j.biopsych.2017.02.1182, PMID: 28410627

[13] Best KP Sullivan T Palmer D Gold M Kennedy DJ Martin J. Prenatal fish oil supplementation and allergy: 6-year follow-up of a randomized controlled trial. Pediatrics. (2016) 137:e20154443. doi: 10.1542/peds.2015-4443, PMID: 27225316

[14] Acevedo N Alashkar Alhamwe B Caraballo L Ding M Ferrante A Garn H. Perinatal and early-life nutrition, epigenetics, and allergy. Nutrients. (2021) 13:724. doi: 10.3390/nu13030724, PMID: 33668787 PMC7996340

[15] Acevedo N Frumento P Harb H Alashkar Alhamwe B Johansson C Eick L. Histone acetylation of immune regulatory genes in human placenta in association with maternal intake of olive oil and fish consumption. Int *J Mol Sci*. (2019) 20:1060. doi: 10.3390/ijms20051060, PMID: 30823645 PMC6429118

[16] Harb H Irvine J Amarasekera M Hii CS Kesper DA Ma Y. The role of PKCζ in cord blood T-cell maturation towards Th1 cytokine profile and its epigenetic regulation by fish oil. Biosci Rep. (2017) 37:BSR20160485. doi: 10.1042/BSR20160485 28159873 PMC5482199

[17] Uauy RD Birch DG Birch EE Tyson JE Hoffman DR. Effect of dietary omega-3 fatty acids on retinal function of very-low-birth-weight neonates. Pediatr Res. (1990) 28:485–92. doi: 10.1203/00006450-199011000-00014, PMID: 2255573

[18] Birch DG Birch EE Hoffman DR Uauy RD. Retinal development in very-low-birth-weight infants fed diets differing in omega-3 fatty acids. Invest Opht Vis Sci. (1992) 33:2365–76. PMID: 1386065

[19] Carlson SE Werkman SH Peeples JM Wilson WM. Long-chain fatty acids and early visual and cognitive development of preterm infants. Eur J Clin Nutr. (1994) 48:S27–30. PMID: 7995262

[20] Dunstan JA Simmer K Dixon G Prescott SL. Cognitive assessment of children at age 2(1/2) years after maternal fish oil supplementation in pregnancy: a randomised controlled trial. Arch Dis Child Fetal Neonatal Ed. (2008) 93:F45–50. doi: 10.1136/adc.2006.099085, PMID: 17185423

[21] Su HM Moser AB Moser HW Watkins PA. Peroxisomal straight-chain acyl-CoA oxidase and D-bifunctional protein are essential for the retroconversion step in docosahexaenoic acid synthesis. J Biol Chem. (2001) 276:38115–20. doi: 10.1074/jbc.M106326200, PMID: 11500517

[22] Helland IB Saugstad OD Saarem K Van Houwelingen AC Nylander G Drevon CA. Supplementation of n-3 fatty acids during pregnancy and lactation reduces maternal plasma lipid levels and provides DHA to the infants. J Matern Fetal Neonat Med. (2006) 19:397–406. doi: 10.1080/14767050600738396 16923694

[23] de Groot RHM Hornstra G van Houwelingen AC Roumen F. Effect of alpha-linolenic acid supplementation during pregnancy on maternal and neonatal polyunsaturated fatty acid status and pregnancy outcome. Am J Clin Nutr. (2004) 79:251–60. doi: 10.1093/ajcn/79.2.251 14749231

[24] Bernard JY De Agostini M Forhan A De Lauzon-Guillain B Charles M-A Heude B. The dietary n6:n3 fatty acid ratio during pregnancy is inversely associated with child neurodevelopment in the EDEN mother–child cohort. J Nutr. (2013) 143:1481–8. doi: 10.3945/jn.113.178640 23902952

[25] Sioen I van Lieshout L Eilander A Fleith M Lohner S Szomner A. Systematic review on n-3 and n-6 polyunsaturated fatty acid intake in European countries in light of the current recommendations – focus on specific population groups. Ann Nutr Metab. (2017) 70:39–50. doi: 10.1159/000456723, PMID: 28190013 PMC5452278

[26] Al MD van Houwelingen AC Kester AD Hasaart TH De Jong AE Hornstra G. Maternal essential fatty acid patterns during normal pregnancy and their relationship to the neonatal essential fatty acid status. Br J Nutr. (1995) 74:55–68. doi: 10.1079/BJN19950106, PMID: 7547829

[27] Otto SJ Houwelingen AC Antal M Manninen A Godfrey K Lopez-Jaramillo P. Maternal and neonatal essential fatty acid status in phospholipids: an international comparative study. Eur J Clin Nutr. (1997) 51:232–42. doi: 10.1038/sj.ejcn.1600390, PMID: 9104573

[28] Hornstra G Al MD van Houwelingen AC Foreman-van Drongelen MM. Essential fatty acids in pregnancy and early human development. Eur J Obstet Gynecol Reprod Biol. (1995) 61:57–62. doi: 10.1016/0028-2243(95)02153-J, PMID: 8549848

[29] Anon. Fats and fatty acids in human nutrition. Ann Nutr Metab. (2009) 55:5–300. 10.1159/000228993 19953704

[30] Agence nationale de sécurité sanitaire de l’alimentation, de l’environnement et du travail. Actualisation des apports nutritionnels conseillés pour les acides gras – Rapport d’expertise collective. Maisons-Alfort: ANSES (2011).

[31] US food and drug administration (FDA) and Environmental Protection Agency (EPA) advisory. Available at: https://www.epa.gov/choose-shand-shellsh-wisely/epa-fda-adviceabout-eating-sh-and-shellsh

[32] European Food Safety Authority (EFSA). EFSA panel on dietetic products, nutrition and allergies (NDA); scientific opinion on the tolerable upper intake level of eicosapentaenoic acid (EPA), docosahexaenoic acid (DHA) and docosapentaenoic acid (DPA); Parma, Italy. EFSA J. (2012) 10:2815. 10.2903/j.efsa.2012.2815 PMC13093066 42016086

[33] Hooperton KE Trépanier MO James NCE Chouinard-Watkins R Bazinet RP. Fish oil feeding attenuates neuroinflammatory gene expression without concomitant changes in brain eicosanoids and docosanoids in a mouse model of Alzheimer’s disease. Brain Behav Immun. (2018) 69:74–90. doi: 10.1016/j.bbi.2017.11.002 29109025

[34] Olsen SF Sorensen JD Secher NJ Hedegaard M Brink Henriksen T Hansen HS. Randomised controlled trial of effect of fish-oil supplementation on pregnancy duration. Lancet. (1992) 339:1003–7. doi: 10.1016/0140-6736(92)90533-9, PMID: 1349049

[35] Helland IB Saugstad OD Smith L Saarem K Solvoll K Ganes T. Similar effects on infants of n-3 and n-6 fatty acids supplementation to pregnant and lactating women. Pediatrics. (2001) 108:e82. doi: 10.1542/peds.108.5.e82, PMID: 11694666

[36] Helland IB Smith L Saarem K Saugstad OD Drevon CA. Maternal supplementation with very-long-chain n-3 fatty acids during pregnancy and lactation augments children’s IQ at 4 years of age. Pediatrics. (2003) 111:e39–44. doi: 10.1542/peds.111.1.e39, PMID: 12509593

[37] Jacobson JL Jacobson SW Muckle G Kaplan-Estrin M Ayotte P Dewailly E. Beneficial effects of a polyunsaturated fatty acid on infant development: evidence from the Inuit of Arctic Quebec. J Pediatr. (2008) 152:356–64. doi: 10.1016/j.jpeds.2007.07.008, PMID: 18280840

[38] Department of Health. Clinical Practice Guidelines: Pregnancy Care. Canberra: Australian Government, Department of Health (2020).

[39] Strauss O. The retinal pigment epithelium in visual function. Physiol Rev. (2005) 85:845–81. doi: 10.1152/physrev.00021.2004, PMID: 15987797

[40] Jacobson TA Glickstein SB Rowe JD Soni PN. Effects of eicosapentaenoic acid and docosahexaenoic acid on low-density lipoprotein cholesterol and other lipids: a review. J Clin Lipidol. (2012) 6:5–18. doi: 10.1016/j.jacl.2011.10.018. Epub 2011 Nov 3, PMID: 22264569

[41] Micallef MA Garg ML. The lipid-lowering effects of phytosterols and (n-3) polyunsaturated fatty acids are synergistic and complementary in hyperlipidemic men and women. J Nutr. (2008) 138:1086–90. doi: 10.1093/jn/138.6.1086, PMID: 18492838

[42] Bahety P Van Nguyen TH Hong Y Zhang L Chan ECY Rache L. Understanding the cholesterol metabolism-perturbing effects of docosahexaenoic acid by gas chromatography–mass spectrometry targeted metabolomic profiling. Europ J Nutr. (2017) 56:29–43. doi: 10.1007/s00394-015-1053-4 26428672

[43] Albert A Alexander D Boesze-Battaglia K. Cholesterol in the rod outer segment: a complex role in a "simple" system. Chem Phys Lipids. (2016) 199:94–105. doi: 10.1016/j.chemphyslip.2016.04.008, PMID: 27216754

[44] Bazan NG. Docosanoids and elovanoids from omega-3 fatty acids are pro-homeostatic modulators of inflammatory responses, cell damage and neuroprotection. Mol Asp Med. (2018) 64:18–33. doi: 10.1016/j.mam.2018.09.003, PMID: 30244005 PMC6204315

[45] Tachikawa M Akanuma SI Imai T Okayasu S Tomohiro T Hatanaka Y. Multiple cellular transport and binding processes of Unesterified docosahexaenoic acid in outer blood-retinal barrier retinal pigment epithelial cells. Biol Pharm Bull. (2018) 41:1384–92. doi: 10.1248/bpb.b18-00185, PMID: 30175775

[46] Nguyen LN Ma D Shui G Wong P Cazenave-Gassiot A Zhang X. Mfsd2a is a transporter for the essential omega-3 fatty acid docosahexaenoic acid. Nature. (2014) 509:503–6. doi: 10.1038/nature13241, PMID: 24828044

[47] Ben-Zvi A Lacoste B Kur E Andreone BJ Mayshar Y Yan H. Mfsd2a is critical for the formation and function of the blood-brain barrier. *Natur*e. (2014) 509:507–11. doi: 10.1038/nature13324, PMID: 24828040 PMC4134871

[48] Wong BH Chan JP Cazenave-Gassiot A Poh RW Foo JC Galam DL. Mfsd2a is a transporter for the essential omega-3 fatty acid docosahexaenoic acid (DHA) in eye and is important for photoreceptor cell development. J Biol Chem. (2016) 291:10501–14. doi: 10.1074/jbc.M116.721340, PMID: 27008858 PMC4865901

[49] Zlokovic BV. The blood-brain barrier in health and chronic neurodegenerative disorders. Neuron. (2008) 57:178–201. doi: 10.1016/j.neuron.2008.01.003, PMID: 18215617

[50] Chow BW Gu C. Gradual suppression of transcytosis governs functional blood-retinal barrier formation. Neuron. (2017) 93:1325–1333.e3. doi: 10.1016/j.neuron.2017.02.043, PMID: 28334606 PMC5480403

[51] Andreone BJ Chow BW Tata A Lacoste B Ben-Zvi A Bullock K. Blood-brain barrier permeability is regulated by lipid transport-dependent suppression of Caveolae-mediated transcytosis. Neuron. (2017) 94:581–594.e5. doi: 10.1016/j.neuron.2017.03.043, PMID: 28416077 PMC5474951

[52] Yang Y-R Xiong X-Y Liu J Wu L-R Zhong Q Zhou K. Mfsd2a (Major facilitator superfamily domain containing 2a) attenuates intracerebral hemorrhage-induced blood-brain barrier disruption by inhibiting vesicular transcytosis. J Am Heart Assoc. (2017) 19:e005811. doi: 10.1161/JAHA.117.005811 PMC5586300 28724654

[53] Ungaro F Tacconi C Massimino L Corsetto PA Correale C Fonteyne P. MFSD2A promotes endothelial generation of inflammation-resolving lipid mediators and reduces colitis in mice. Gastroenterol. (2017) 153:1363–1377.e6. doi: 10.1053/j.gastro.2017.07.048, PMID: 28827082

[54] Macura JI Djuricic I Major T Milanovic D Brkic B Sobajic S. The high-dose fish oil supplementation increased Mfsd2a expression without altering DHA levels in the healthy retina. J Funct Foods. (2022) 99:105302. doi: 10.1016/j.jff.2022.105302

[55] Kates M. Techniques in lipidology: Isolation, analysis and identification of lipids. 3rd ed. Amsterdam: North-Holland Publishing Company (1972).

[56] Ichihara K Fukubayashi Y. Preparation of fatty acid methyl esters for gas-liquid chromatography. J Lipid Res. (2010) 51:635–40. doi: 10.1194/jlr.D001065, PMID: 19759389 PMC2817593

[57] Livak KJ Schmittgen TD. Analysis of relative gene expression data using real-time quantitative PCR and the 2(-Delta Delta C(T)). Method. (2001) 25:402–8. doi: 10.1006/meth.2001.1262, PMID: 11846609

[58] Rice DS Calandria JM Gordon WC Jun B Zhou Y Gelfman CM. Adiponectin receptor 1 conserves docosahexaenoic acid and promotes photoreceptor cell survival. Nat Commun. (2015) 6:6228. doi: 10.1038/ncomms7228 25736573 PMC4351799

[59] Zhang CL Wang HL Li PC Hong CD Chen AQ Qiu YM. Mfsd2a overexpression alleviates vascular dysfunction in diabetic retinopathy. Pharmacol Res. (2021) 171:105755. doi: 10.1016/j.phrs.2021.105755, PMID: 34229049

[60] Horton JD. Sterol regulatory element-binding proteins: transcriptional activators of lipid synthesis. Biochem Soc Transac. (2002) 30:1091–5. doi: 10.1042/bst0301091, PMID: 12440980

[61] Lobanova ES Schuhmann K Finkelstein S Lewis TR Cady MA Hao Y. Disrupted blood-retina lysophosphatidylcholine transport impairs photoreceptor health but not visual signal transduction. J Neurosci. (2019) 39:9689–701. doi: 10.1523/JNEUROSCI.1142-19.2019, PMID: 31676603 PMC6891062

[62] Kalaany NY Mangelsdorf DJ. LXRs and FXR: the yin and yang of cholesterol and fat metabolism. Annu Rev Physiol. (2006) 68:159–91. doi: 10.1146/annurev.physiol.68.033104.152158, PMID: 16460270

[63] Siperstein MD Fagan VM. Feedback control of mevalonate synthesis by dietary cholesterol. J Biol Chem. (1966) 241:602–9. doi: 10.1016/S0021-9258(18)96879-1, PMID: 5908125

[64] Mauch DH Nagler K Schumacher S Goritz C Muller EC Otto A. CNS synaptogenesis promoted by glia-derived cholesterol. Science. (2001) 294:1354–7. doi: 10.1126/science.294.5545.1354, PMID: 11701931

[65] Lund EG Guileyardo JM Russell DW. cDNA cloning of cholesterol 24-hydroxylase, a mediator of cholesterol homeostasis in the brain. Proc Natl Acad Sci U S A. (1999) 96:7238–43. doi: 10.1073/pnas.96.13.7238, PMID: 10377398 PMC22064

[66] Cali JJ Hsieh CL Francke U Russell DW. Mutations in the bile acid biosynthetic enzyme sterol 27-hydroxylase underlie cerebrotendinous xanthomatosis. J Biol Chem. (1991) 266:7779–83. doi: 10.1016/S0021-9258(20)89518-0, PMID: 2019602 PMC4449724

[67] Moriguchi T Harauma A Salem N Jr. Plasticity of mouse brain docosahexaenoic acid: modulation by diet and age. Lipids. (2013) 48:343–55. doi: 10.1007/s11745-013-3775-5, PMID: 23460301

[68] Zheng W Reem RE Omarova S Huang S DiPatre PL Charvet CD. Spatial distribution of the pathways of cholesterol homeostasis in human retina. PLoS One. (2012) 7:e37926. doi: 10.1371/journal.pone.0037926, PMID: 22629470 PMC3358296

[69] Chen CT Kitson AP Hopperton KE Domenichiello AF Trepanier M-O Lin LE. Plasma non-esterified docosahexaenoic acid is the major pool supplying the brain. Sci Rep. (2015) 5:15791. doi: 10.1038/srep15791, PMID: 26511533 PMC4625162

[70] Simón MV Agnolazza DL German OL Garelli A Politi LE Agbaga MP. Synthesis of docosahexaenoic acid from eicosapentaenoic acid in retina neurons protects photoreceptors from oxidative stress. J Neurochem. (2016) 136:931–46. doi: 10.1111/jnc.13487, PMID: 26662863 PMC4755815

[71] Igarashi M Ma K Chang L Bell JM Rapoport SI. Dietary n-3 PUFA deprivation for 15 weeks upregulates elongase and desaturase expression in rat liver but not brain. J Lipid Res. (2007) 48:2463–70. doi: 10.1194/jlr.M700315-JLR200 17715424

[72] Scott BL Bazan NG. Membrane docosahexaenoate is supplied to the developing brain and retina by the liver. Proc Natl Acad Sci U S A. (1989) 86:2903–7. doi: 10.1073/pnas.86.8.2903, PMID: 2523075 PMC287028

[73] Rapoport SI Rao JS Igarashi M. Brain metabolism of nutritionally essential polyunsaturated fatty acids depends on both the diet and the liver. Prostaglandins Leukot Essent Fat Acids. (2007) 77:251–61. doi: 10.1016/j.plefa.2007.10.023, PMID: 18060754 PMC2725409

[74] German OL Insua MF Gentili C Rotstein NP Politi LE. Docosahexaenoic acid prevents apoptosis of retina photoreceptors by activating the ERK/MAPK pathway. J Neurochem. (2006) 98:1507–20. doi: 10.1111/j.1471-4159.2006.04061.x, PMID: 16923163

[75] German OL Monaco S Agnolazza DL Rotstein NP Politi LE. Retinoid X receptor activation is essential for docosahexaenoic acid protection of retina photoreceptors. J Lipid Res. (2013) 54:2236–46. doi: 10.1194/jlr.M039040, PMID: 23723389 PMC3708373

[76] Chan JP Wong BH Chin CF Galam DLA Foo JC Wong LC. The lysolipid transporter Mfsd2a regulates lipogenesis in the developing brain. *PLoS Bio*l. (2018) 724:e2006443. doi: 10.1371/journal.pbio.2006443 PMC6093704 30074985

[77] Macura JI Zivanovic A Perovic M Ciric J Major T Kanazir S. The expression of Major facilitator superfamily domain-containing Protein2a (Mfsd2a) and aquaporin 4 is altered in the retinas of a 5xFAD mouse model of Alzheimer's disease. Int J Mol Sci. (2023) 24:14092. doi: 10.3390/ijms241814092, PMID: 37762391 PMC10531902

[78] Le Jossic-Corcos C Gonthier C Zaghini I Logette E Shechter I Bournot P. Hepatic farnesyl diphosphate synthase expression is suppressed by polyunsaturated fatty acids. Biochem J. (2005) 385:787–94. doi: 10.1042/BJ20040933 15473864 PMC1134755

[79] Boergesen M Pedersen TA Gross B van Heeringen SJ Hagenbeek D Bindesboll C. Genome-wide profiling of liver X receptor, retinoid X receptor, and peroxisome proliferator-activated receptor alpha in mouse liver reveals extensive sharing of binding sites. Mol Cell Biol. (2012) 32:852–67. doi: 10.1128/MCB.06175-11, PMID: 22158963 PMC3272984

[80] Chen W Jump DB Esselman WJ Busik JV. Inhibition of cytokine signaling in human retinal endothelial cells through modification of caveolae/lipid rafts by docosahexaenoic acid. Invest Ophthal Vis Sci. (2007) 48:18–73426. doi: 10.1167/iovs.06-0619, PMID: 17197511 PMC1975816

[81] Wang Z Liu CH Huang S Fu Z Tomita Y Britton WR. Wnt signaling activates MFSD2A to suppress vascular endothelial transcytosis and maintain blood-retinal barrier. Sci Adv. (2020) 6:eaba7457. doi: 10.1126/sciadv.aba7457 32923627 PMC7455181

[82] Zhao WN Hylton NK Wang J Chindavong PS Alural B Kurtser I. Activation of WNT and CREB signaling pathways in human neuronal cells in response to the Omega-3 fatty acid docosahexaenoic acid (DHA). Mol Cell Neurosci. (2019) 99:103386. doi: 10.1016/j.mcn.2019.06.006, PMID: 31202891 PMC7001743

[83] Buccitelli C Selbach M. mRNAs, proteins and the emerging principles of gene expression control. Nat Rev Genet. (2020) 21:630–44. doi: 10.1038/s41576-020-0258-4, PMID: 32709985

[84] Wolf J Lapp T Reinhard T Agostini H Schlunck G Lange C. Webbasierte Genexpressionsanalysen – auf dem Weg zur molekularen Entschlüsselung gesunder und erkrankter Augengewebe [web-based gene expression analysis-paving the way to decode healthy and diseased ocular tissue]. Fortschr Ophthalmol. (2022) 119:929–36. doi: 10.1007/s00347-022-01592-9, PMID: 35194679 PMC8863098

[85] Choi YK Kim JH Kim WJ Lee HY Park JA Lee S-W. AKAP12 regulates human blood-retinal barrier formation by downregulation of hypoxia-inducible Factor-1. J Neurosci. (2007) 27:4472–81. doi: 10.1523/JNEUROSCI.5368-06.2007, PMID: 17442832 PMC6672308

[86] Chen J Stahl A Krah NM Seaward MR Dennison RJ Sapieha P. Wnt signaling mediates pathological vascular growth in proliferative retinopathy. Circulation. (2011) 124:1871–81. doi: 10.1161/CIRCULATIONAHA.111.040337, PMID: 21969016 PMC3326389

[87] Luo Y Xiao W Zhu X Mao Y Liu X Chen X. Differential expression of Claudins in retinas during Normal development and the angiogenesis of oxygen-induced retinopathy. Invest Opthal Vis Sci. (2011) 52:7556. doi: 10.1167/iovs.11-7185, PMID: 21862644

[88] Strong S Liew G Michaelides M. Retinitis pigmentosa-associated cystoid macular oedema: pathogenesis and avenues of intervention. Brit J Ophthal. (2017) 101:31–7. doi: 10.1136/bjophthalmol-2016-309376 27913439 PMC5256121

[89] Klaassen I Van Noorden CJF Schlingemann RO. Molecular basis of the inner blood-retinal barrier and its breakdown in diabetic macular edema and other pathological conditions. *Progr Ret Eye* Res. (2013) 34:19–48. doi: 10.1016/j.preteyeres.2013.02.001, PMID: 23416119

