# Supplementary figures and images for: The supplementation of a high dose of fish oil during pregnancy and lactation led to an elevation in Mfsd2a expression without any changes in docosahexaenoic acid levels in the retina of healthy 2-month-old mouse offspring
Source: Front Nutr. 2024 Jan 24;10:1330414. doi: 10.3389/fnut.2023.1330414 (PMC10847253; doi:10.3389/fnut.2023.1330414)

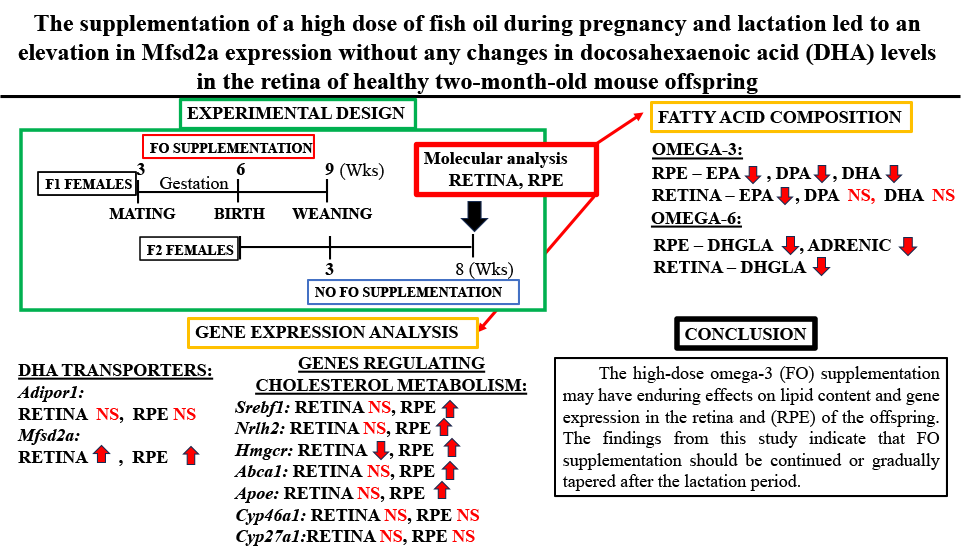

Supplement: Supplementary file 1 [file Image_1.TIF]
